# Impact of breast cancer stage, time from diagnosis and chemotherapy on plasma and cellular biomarkers of hypercoagulability

**DOI:** 10.1186/1471-2407-14-991

**Published:** 2014-12-22

**Authors:** Mourad Chaari, Ines Ayadi, Aurelie Rousseau, Eleftheria Lefkou, Patrick Van Dreden, Fatoumata Sidibe, Hela Ketatni, Vassiliki Galea, Amir Khaterchi, Racem Bouzguenda, Mounir Frikha, Lilia Ghorbal, Jamel Daoud, Choumous Kallel, Martin Quinn, Joseph Gligorov, Jean Pierre Lotz, Mohamed Hatmi, Ismail Elalamy, Grigoris T Gerotziafas

**Affiliations:** Service d’Hématologie Biologique Hôpital Tenon, Hôpitaux Universitaires de l’Est Parisien, Assistance Publique Hôpitaux de Paris, Paris, France; Laboratoire d’Hématologie, Hôpital universitaire Habib Bourguiba, Sfax, Tunisia; Service de Carcinologie, Hôpital Universitaire Habib Bourguiba, Sfax, Tunisia; INSERM U938, Faculté de Médecine Pierre et Marie Curie, Université Paris VI, Paris, France; Research and Development, Diagnostica Stago, Gennevilliers, France; Service de Radiothérapie Carcinologique, Hôpital Universitaire Habib Bourguiba, Sfax, Tunisia; Service d’Oncologie Médicale et de Thérapie Cellulaire, Hôpitaux Universitaires de l’Est-Parisien, Institut Universitaire de Cancérologie, Faculté de Médecine Pierre et Marie Curie, Université Paris VI, APREC, Paris, France; Département Infection et Epidémiologie, Institut Pasteur, Paris, France

**Keywords:** Breast cancer, Venous thromboembolism, Thrombin generation, Microparticles, D-Dimers, Risk assessment model

## Abstract

**Background:**

In breast cancer patients routine thromboprophylaxis is not recommended but individualized risk assessment is encouraged. The incorporation of hypercoagulability biomarkers could increase the sensitivity of risk assessment models (RAM) to identify patients at VTE risk. To this aim we investigated the impact of cancer-related characteristics on hypercoagulability biomarkers.

**Methods:**

Thrombin generation (TG) assessed with the Thrombogramme-Thrombinoscope®, levels of platelet derived microparticles (Pd-MP) assessed with flow cytometry, procoagulant phospholid dependent clotting time (PPL-ct) measured with a clotting assay and D-Dimers (were assessed in a cohort of 62 women with breast cancer and in 30 age matched healthy women.

**Results:**

Patients showed significantly higher TG, Pd-MP, D-Dimers levels and shortened PPL-ct compared to the controls. The PPL-ct was inversely correlated with the levels of Pd-MP, which were increased in 97% of patients. TG and D-Dimers were increased in 76% and 59% of patients respectively. In any stage of the disease TG was significantly increased as compared to the controls. There was no significant difference of TG in patients with local, regional of metastatic stage. There was no significant difference in Pd-MP or Pd-MP/PS^+^ between the subgroups of patients with local or regional stage of cancer. Patients with metastatic disease had significantly higher levels of Pd-MP and Pd-MP/PS^+^ compared to those with regional stage. The D-Dimers increased in patients with metastatic stage. In patients on chemotherapy with less than 6 months since diagnosis TG was significantly higher compared to those on chemotherapy who diagnosed in interval > 6 months. Patients with metastatic disease had significantly higher levels of Pd-MP and D-Dimers compared to those with non-metastatic disease.

**Conclusion:**

In breast cancer patients the stage, the time elapsed since the diagnosis and the administration of chemotherapy are determinants of cellular and plasma hypercoagulability. The levels and the procoagulant activity of Pd-MP are interconnected with the biological activity and the overall burden of cancer. TG reflects the procoagulant properties of both breast cancer and chemotherapy in the initial period of cancer diagnosis. Thus the weighted incorporation of the biomarkers of cellular and plasma hypercoagulabilty in RAM for VTE might improve their predictive value.

## Background

The close association of cancer with hypercoagulability and the risk of thrombosis have been recognized since the 19th century [1–3]. The risk of venous thromboembolism (VTE) is about 7-fold higher in cancer patients compared to controls [4, 5]. VTE significantly affects morbidity and is the second cause of mortality in hospitalized cancer patients [6–9]. Many aspects of the interplay between cancer and blood coagulation have been elucidated by experimental, clinical and epidemiological studies [10, 11]. The histological type, the burden of cancer cells, the stage of the disease, the use of chemotherapy and the time since diagnosis are determinants of the VTE risk [12].

Breast cancer is the commonest malignancy in women and is considered to be associated with low VTE risk as compared to other malignancies. In women with newly diagnosed breast cancer the cumulative incidence of VTE is less than 1% [10, 12]. However VTE risk increases by 4- to 6-fold during chemotherapy as well as in advanced stage or metastatic disease [13]. Routine administration of thromboprophylaxis is not recommended in women with breast cancer undergoing adjuvant chemotherapy since there are no relevant clinical trials assessing the efficacy and safety of antithrombotic agents in this context [14]. However, expert consensus statements encourage an individualized approach for the identification of patients at risk of VTE who are eligible for pharmacological thromboprophylaxis [15]. To this aim, Korhana et al. have developed and prospectively validated a risk assessment model that stratifies cancer patients to high, moderate or low risk for VTE prior to chemotherapy initiation [16].

Thrombosis is a multifactorial disease occurring when the Virchow’s triade (blood hypercoagulability, vessel wall lesion and alteration of blood flow) is fulfilled. However, current risk assessment models for VTE in cancer patients are restricted to some clinical risk factors and are missing the evaluation of blood borne hypercoagulability, although this is one of the basic components of Virchow’s triad. The expression of tissue factor (TF) by cancer cells as well as the formation of procoagulant microparticles derived from activated platelets, are pivotal events leading to enhanced thrombin generation in patients with cancer (reviewed in [17–20]). TF-induced activation of blood coagulation in cancer patients leads to sustained thrombin generation and fibrin formation [21]. The D-Dimers are degradation products of cross-linked fibrin, indicating either enhanced fibrin formation or activation of the fibrinolytic system, or increased levels of fibrinogen and likely reflect the biological activity of cancer cells [22]. Increased concentration of D-Dimers in plasma has been observed in patients with breast, prostate or bowel cancer [23].

It has been reported that incorporation of biomarkers of cellular or plasma hypercoagulability increases the sensitivity of the risk assessment models to identify cancer patients at risk for VTE [24]. The aim of the present study was to investigate the potential relation between cancer-related characteristics and the biomarkers of plasma and cellular hypercoagulability. The capacity of thrombin generation in patients’ plasma, the concentration of procoagulant platelet-derived microparticles expressing phosphatidylserin (Pd-MP/PS^+^) in plasma, the procoagulant phospholid (PPL) dependent clotting time and D-Dimers were assessed in a cohort of women suffering from breast cancer. These biomarkers of plasma and cellular hypercoagulability were analyzed in relation to the stage of the disease, the time elapsed since diagnosis and the administration of chemotherapy.

## Methods

### Cancer patients

Out-patients with histologically proven breast cancer were enrolled in the study from January to June 2012. Patients were considered under chemotherapy if they had received a chemotherapy cycle 21 days earlier. The exclusion criteria were: age less than 18 years, recent (<6 months) documented episode of VTE (deep venous thrombosis and/or pulmonary embolism) or acute coronary syndrome, confirmed pregnancy, major psychiatric disorders, life expectancy less than 3 months, active anticoagulant treatment, recent (<3 months) hospitalization for acute medical illness or major surgery, recent surgery (<2 months).

### Classification of the patients

Patients were classified for post hoc analysis according to the tumor, node, metastases (TNM) system of stratification: Local stage was defined by the absence of axillary nodes and distant metastasis (TxN0M0). Regional stage was defined by the presence of axillary node(s) and the absence of distant metastasis (TxN + M0). The metastasis stage was defined by the presence of one or more distant metastases (TxNxM+) [25]. Patients were also stratified according to the presence or not of at least one cardiovascular risk factor. Stratification according to hormone positive or negative receptor breast carcinoma was not possible since data were not available for all patients.

### Control group

The control group consisted of 30 age-matched healthy women who did not have breast cancer and who were not taking any medication for at least one month before blood sampling. Healthy volunteers had normal prothrombin time (PT) and activated partial thromboplastin time (aPTT) and had no personal history of thrombotic or hemorrhagic episodes. The values obtained in this population, comparable in age to the breast cancer patients, were used to establish reference intervals for the assays. All patients and healthy individuals gave written informed consent for participation in the study.

### Blood samples

Blood samples were obtained by traumatic puncture of the antecubital vein, using a 20-gauge needle, and placed into siliconized vacutainer tubes containing 0.129 mol/L trisodium citrate (from Becton and Dickinson France) as anticoagulant, in a ratio of nine parts of blood to one part of citrate. Platelet poor plasma (PPP) was obtained after double centrifugation of citrated whole blood for 20 minutes at 2000 *g*. Platelet-free plasma was prepared immediately after blood sampling using a 2-step centrifugation procedure: initially at 1500 *g* for 15 minutes at 20°C to prepare platelet rich plasma and then at 13000 *g* for 2 minutes at 20°C to prepare PFP. Samples were aliquoted and frozen at −80°C until assayed. All measurements were done in thawed plasma samples. All PPP samples were from vein punctures performed for routine evaluation of blood coagulation tests. Blood anticoagulated with EDTA was used for the determination of complete blood count. This study was approved by the ethics committee of Tenon University Hospital and was performed in accordance with the principles embodied in the Declaration of Helsinki.

### Thrombin generation in plasma

Thrombin generation in PPP was assessed using the Calibrated Automated Thrombogram assay (CAT®, Diagnostica Stago, France) as described by Hemker et al. [26]. Briefly 80 μl of PPP was added to 20 μl of PPP-reagent 5 pM® (Thrombinoscope b.v., Maastricht, Netherlands), that is a mixture of TF (5 pM final concentration in plasma) and phospholipids (4 μM final concentration in plasma). Each patient’s plasma was studied in duplicate. In a third well, PPP reagent 5 pM® was replaced with the same volume of Thrombin Calibrator® (Thrombinoscope bv, Maastricht, Netherlands) to correct thrombin generation curves for substrate consumption and the inner filter fluorescence effects. Thrombin generation was triggered with a 20 μl solution containing CaCl_2_ (16.7 mM final concentration) and the fluorogenic substrate Z-Gly-Gly-Arg-AMC (417 pM final concentration). Fluorescence was measured using a Fluoroscan Ascent®fluorometer (ThermoLabsystems, Helsinki, Finland). Acquisition of thrombin generation parameters was performed using the appropriate software (Calibrated Automated Thrombogram®bv, Maastricht, Netherlands). Among thrombogram parameters we analyzed the endogenous thrombin potential (ETP) that reflects the integral thrombin activity, the Peak concentration of thrombin and the mean rate index (MRI), which reflects the rate of the propagation phase of thrombin generation [calculated by the formula MRI = Peak/(ttPeak – lag-time)].

### Microparticle labelling and flow cytometry analysis

Platelet-derived microparticles were measured in platelet free plasma using a flow cytometry assay as described by Robert et al. [27]. Briefly, for Pd-MP/PS^+^ labelling, 30 μL of fresh PFP was incubated with 10 μL of a solution of phycoerythrin (PE) bound monoclonal antibody against platelet glycoprotein IIb (CD41). For the detection of phosphatidylserine expression by Pd-MP the plasma samples were additionally spiked with 10 μL of fluoresce in isothiocyanate (FITC) labelled recombinant human nnexin V. Anti-CD41 monoclonal antibody was purchased from BioCytex (Marseille, France). Human annexin -FITC kit was obtained from AbCys (Paris, France). Concentration-matched isotype antibodies (IgG_1_–PE, 15 ng/μL, clone 2DNP-2H11, from BioCytex) or Annexin V-FITC with phosphate-buffered saline without calcium were used as controls. Analyses were performed on Cytomics FC500 flow cytometer (Beckman-Coulter, Villepinte, France). To limit background noise from dust and crystals, the instrument was operated using a 0.22 μm filtered sheath fluid (IsoflowTM; Beckman-Coulter, France). The software packages CXP ACQUISITION® and CXP ANALYSIS® (Beckman-Coulter, France) were used for data acquisition and analysis, respectively. Standardization of the Pd-MP protocol was done using a blend of mono-disperse fluorescent beads (Megamix, BioCytex Marseille, France) of three diameters (0.5, 0.9 and 3 μm). Forward scatter and side scatter parameters were plotted on logarithmic scales to best cover a wide size range. Pd-MP were defined as single positive CD41^+^ events. CD41 positivity was displayed on single parameter histograms. Pd-MP/PS^+^ were defined as dual-positive phosphatidylserine PS^+^/CD41 events as displayed on dual-color fluorescence plots after staining with annexin V-FITC and CD41-PE. In each studied sample 30 μl of counting beads with an established concentration close to 1000 beads/μl (Flow Count^TM^Fluorosphores Beckman-Coulter) were added in order to express counts as absolute numbers of microparticles per microliter of PFP. All plasma samples were assessed for Pd-MP within one week after blood collection and after one cycle of freezing /thawing. Application of the same experimental conditions reduced the impact of the eventual error introduced by the freezing/thawing on Pd-MP concentration.

### Assessment of procoagulant phospholipid dependent activity in plasma

Procoagulant phospholipid-dependent clotting time (PPL) was measured in thawed PPP using the factor Xa - based coagulation assay (PPL clotting time) STA®Procoag-PPL, (DiagnosticaStago, Asnières, France) in which shortened clotting times are associated with increased levels of procoagulant phospholipids. The PPL clotting time was performed according to the manufacturer’s instructions on a STA®-R analyser.

### D-Dimers

The concentration of D-Dimers in platelet poor plasma was determined using the enzyme linked fluorescent assay on a mini VIDAS system (bio-Merieux, Paris, France). The assay employs a quantitative sandwich enzyme immunoassay technique combining a bound anti-D-Dimer monoclonal immunoglobulin with an unbound enzyme labeled anti-D-dimer monoclonal immunoglobulin. Results are reported in ng/mL of fibrinogen equivalent units. According to manufacturer’s instructions, D-Dimers concentrations equal or lower than 500 ng/ml were considered as normal.

### Routine biochemical and hematological assessment

Blood samples were also obtained for the assessment of transaminase levels (ASAT and ALAT), CRP, urea and creatinine. Routine hemogram parameters as well as prothrombin time (expressed as percentage of prothrombin) and activated partial thromboplastin time (expressed as ratio of patients/control values), were also analyzed. Routine hematological and biochemical measurements were performed with standardized assays existing in the central hematological and biochemical hospital laboratory.

### Statistical analysis

The potential changes of the studied biomarkers in the group of breast cancer patients versus the control group as well as in the subgroups of patients stratified according to the stage of the cancer, the chemotherapy and the time since the diagnosis were unknown. Consequently, determination of the sample size according to a power analysis based on the predicted differences of the studied biomarkers in function of cancer related variables was not feasible. For this reason, the sample size for each one of the main groups (patients and controls) and consequently for the subgroups of patients, was based on the minimum number of individuals required in order to apply the statistical tests which were used. Continuous variables are expressed by means ± standard deviation. In the groups of patients and controls comparisons between continuous variables were performed using Student’s *t*-test when they were normally distributed and Mann–Whitney test when they were abnormally distributed and when variables had a coefficient of variation higher than 100%. One way ANOVA test was used to determine the possible differences among subgroups of patients (defined according the stage of cancer the presence of chemotherapy and the time since diagnosis and controls). Homogeneity of the values was tested with Levene test for equality of errors in variances and normality of residues was verified by the Shapiro-Wilk test. The Kruskal-Wallis test was used when no homogeneity was documented. For significant variables post hoc LSD test was applied to compare differences between groups. Multiple comparisons and Spearman coefficient correlations were calculated. When appropriate, the upper and lower normal limits (UNL and LNL respectively) for the studied biomarkers of hypercoagulability were defined respectively as upper and lower limit of the 95% confidence interval (CI) of normal values obtained by performing the corresponding tests in the control group (healthy volunteers). Thrombin generation was considered as increased when at least one of the studied parameters (ETP, Peak or MRI) showed a value higher than the UNL. Two-sided p-value <0.05 was considered significant. Statistical analysis was performed using SPSS 20.0 (SPSS Inc., Chicago, IL).

## Results

### Patients characteristics

A total of 62 women with breast cancer were included in the study. The mean age of the breast cancer group and the control group was not significantly different (52 ± 11 years and 55 ± 10 years respectively; p > 0.05). Basic hematological parameters in the breast cancer group were within the normal range and not significantly different compared to the control group. The body mass index was also not significantly different between the two groups. The CRP levels were above the normal in 8 our 62 patients (12%).

Patients were stratified in subgroups according to the stage of the disease as follows: 13 had a local stage, 29 had a regional stage and 20 had metastatic disease. In the subgroup with metastatic stage disease, 95% had bone metastasis and 40% also had liver or lung metastases. Age, BMI and basic hematological parameters were not significantly different among these subgroups, as well as between each subgroup and the control group. Patients were also stratified to those who were on active chemotherapy (n = 41).

Patients were also stratified according to the time elapsed since the diagnosis of cancer: <6 months (n = 27) and more than 6 months (n = 35; of home 10 patients were diagnosed 6–12 months, 13 patients were diagnosed 12–36 months and 12 patients were diagnosed more than 36 months before the inclusion in the study). Invasive ductal carcinoma of the breast was diagnosed in 90% of patients. Curative surgery was performed in 82% of the patients included in the study. All surgical procedures were completed at least 2 months before enrolment. In 42 out of 62 patients (67%) at least one cardiovascular risk factor was present. Demographic and clinical characteristics of the studied groups are summarized in Table 1.

**Table 1:** **Demographic data, clinical characteristics and routine hematological and biochemical parameters of breast cancer patients and controls**

	Control group (n = 30)	***All patients (n = 62)***	Localized stage (n = 13)	Regional stage (n = 29)	Metastatic stage (n = 20)
Age (year)	55 ± 10	*52 ± 11*	53 ± 10	54 ± 11	49 ± 13
BMI (Kg/m^2^)	27 ± 6	*28 ± 4.7*	29.6 ± 4.7	27.6 ± 4.2	27.7 ± 5.6
Hemoglobin (g/dL)	11.6 ± 2	*11.5 ± 1.5*	11.2 ± 1.1	11.8 ± 1.4	11.3 ± 1.8
Leukocytes (10^9^/L)	7.3 ± 1.8	*7.1 ± 4.8*	7.2 ± 2.4	7.1 ± 2.8	7.1 ± 5
Platelets (10^9^/L)	270 ± 62	*242 ± 96*	262 ± 65	264 ± 98	198 ± 98
PT (% of prothrombin)	100 ± 8	*92 ± 8.3*	94 ± 6	95 ± 7	89 ± 11
aPTT ratio	1 ± 0.2	*0.9 ± 0.06*	0.9 ± 0.05	0.9 ± 0.06	0.9 ± 0.08
Fibrinogen (g/L)	3.9 ± 0.6	*4.4 ± 1.2*	4 ± 0.8	4.7 ± 1.4	4.4 ± 1.1
CRP (mg/L)	-	*14.5 ± 50*	32 ± 99	7 ± 20	10 ± 15
ALAT (IU/L)	-	*25 ± 13*	30 ± 16	24 ± 10	25 ± 15
ASAT (IU/L)	-	*36 ± 23*	31 ± 18	34 ± 17	43 ± 32
Creatinine (μmol/L)	-	*65 ± 17*	67 ± 23	67 ± 17	62 ± 13
Urea (mg/dL)	-	*5 ± 2*	5 ± 1.6	5.5 ± 2.7	5 ± 2.2
**Time since diagnosis (n)**					
0-6 months		*27*	8	15	4
6-12 months		*10*	2	7	1
12-36 months	-	*13*	3	5	5
> 36 months		*12*	0	2	10
**Cardiovascular risk factors (n)**					
-Hypertension	no	*14*	3	9	2
-Varicose veine	no	*8*	1	6	1
-Hyperlipidemia	no	*4*	1	3	0
-Diabetes	no	*6*	1	3	2

Data are presented as mean ± sd.

### Thrombin generation in breast cancer patients

Thrombin generation was significantly increased in breast cancer patients as compared to the control group (Table 2). The MRI was significantly higher in the group of patients as compared to the control group (159 ± 47 nM/min versus 109 ± 33 nM/min respectively; p < 0.001). The Peak was also higher in cancer patients as compared to the control group (341 ± 65 nM versus 288 ± 48 nM, respectively; p = 0.001). The ETP was not significantly different between the cancer group and the control group (1531 ± 337 nM.min versus 1498 ± 225 nM.min).

**Figure 1:**
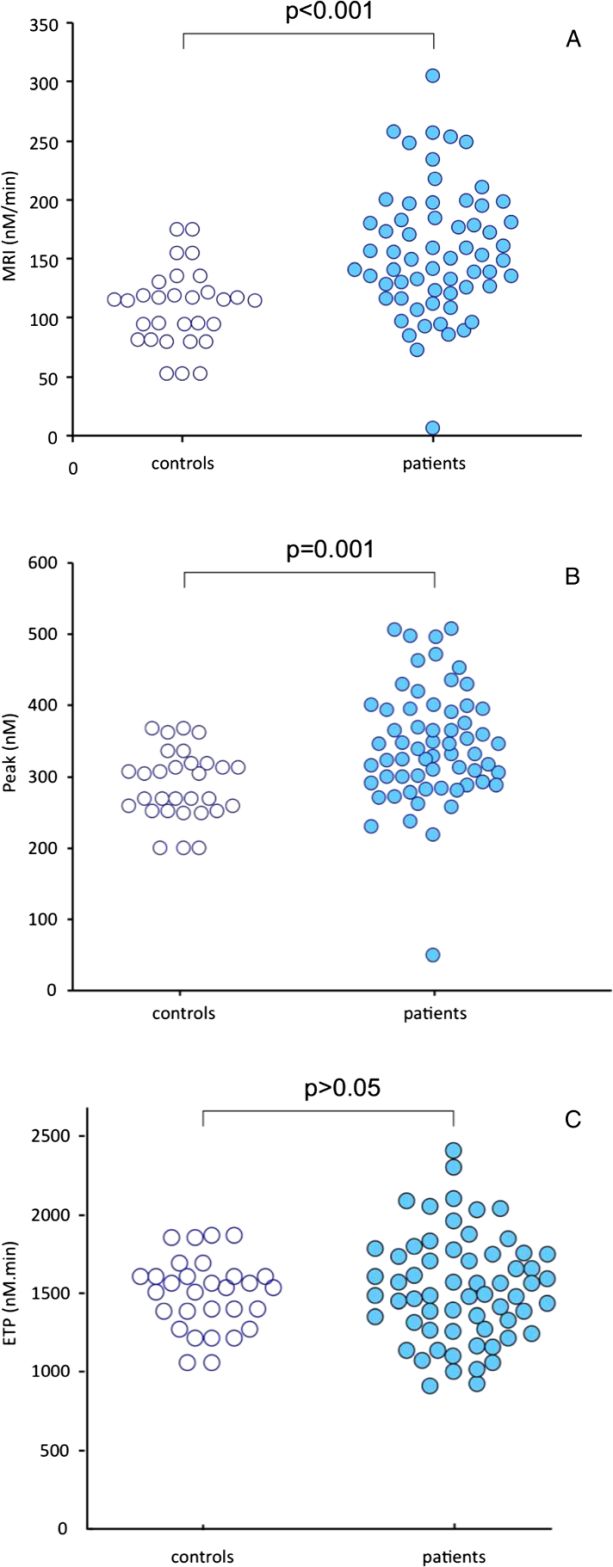
**Distribution of individual values of thrombin generation rate (frame A), Peak of thrombin (frame B) and ETP (frame C) in the control group (open cycles) and in the group of patients (dark cycles).**

**Figure 2:**
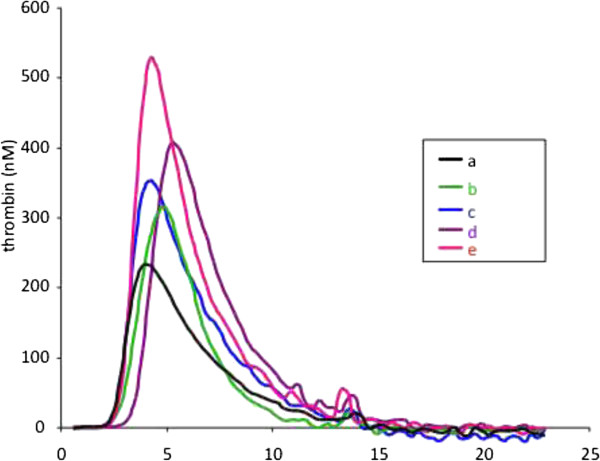
**Representative thrombograms from a healthy individual (a) and four patients with high thrombin generation (b, c, d, e).**

The distribution of the individual values of thrombogram parameters in cancer patients and controls is shown in Figure 1. Representative thrombograms of patients with increased and normal thrombin generation profile are depicted in Figure 2. The MRI was higher than the UNL in 47 patients (76%). The Peak was higher than the UNL in 42 patients (68%). The ETP was higher than the UNL in 25 patients (40%). Among patients with high thrombin generation 21 (33%) had the three parameters of thrombogram (MRI, Peak and ETP) higher than the UNL. In 21 patients (33%) the MRI and the Peak was higher than the UNL.

**Table 2:** **Biomarkers of cellular and plasma hypercoagulability in patients and controls**

	Control group (n = 30) (95% CI)	Breast cancer group (n = 62)			
		All patients (n = 62) (95% CI)	Local stage (n = 13) (95% CI)	Regional stage (n = 29) (95% CI)	Metastatic stage (n = 20) (95% CI)
**MRI** ***(nM/min)***	109 ± 33 (96,5–121)	159 ± 47** (139–168)	172 ± 55^§*^ (138–205)	146 ± 56^§^ (122–168)	162 ± 49** (126–180)
**Peak** ***(nM)***	288 ± 48 (269–305)	341 ± 65* (323–365	369 ± 75** (323–414)	334 ± 90^§§^ (301–371)	343 ± 69^§^ (306–370)
**ETP** ***(nM.min)***	1498 ± 225 (1413–1581)	1531 ± 337 (1448–1623)	1626 ± 332 (1424–1826)	1499 ± 374 (1366–1656)	1515 ± 285 (1369–1650)
**Pd-MP (/μL)**	756 ± 429 (650–1100)	10015 ± 8223^§§^ (7890–12138)	9370 ± 7724 (4702–14038)	7847 ± 6479*** 5334–10359)	13650 ± 9864 (8895–18404)
**Pd-MP/PS** ^**+**^ **(/μL)**	695 ± 361 (550–1020)	9698 ± 7931^§§^ (7649–11746)	9115 ± 7428 (4626–13604)	7570 ± 6241*** (5150–9991)	13231 ± 9512 (8646–17816)
**PPL (sec)**	72.8 ± 9.9 (59–66)	43,5 ± 10,3 (41–46)	45 ± 10,2 (39–51)	44,3 ± 9,8 (40–48)	41,3 ± 11,3^$^ (35–47)
**D-Dimers (ng/ml)**	230 ± 50 (279–340)	1250 ± 1773 (767–1648)	605 ± 499*** (303–907)	1123 ± 1429 (503–1572)	1853 ± 2497 (703–3154)

Values are depicted as mean ± sd. The 95% Confidence Interval of the mean (95% CI) is also shown.

*p = 0,001 versus controls, **p < 0,001 versus controls, ^**§**^p < 0,01 versus controls, ^**§§**^p < 0,05 versus controls, ***p < 0,05 versus metastatic stage, ^$^p < 0,05 versus local stage.

### Influence of stage, time and chemotherapy on thrombin generation

**Figure 3:**
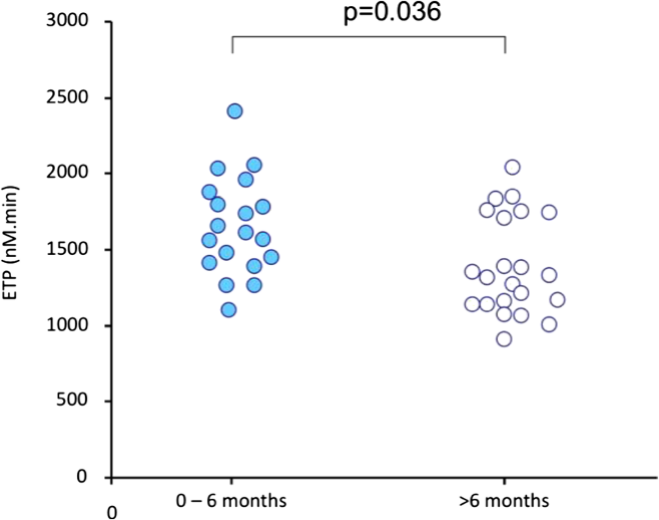
**Impact of the interval since the diagnosis (shorter of longer than 6 months) on the ETP in patients on chemotherapy.**

In any stage of the breast cancer (local, regional and metastatic) thrombogram parameters were significantly increased as compared to the control group (Table 2). Thrombin generation was significantly higher in patients with newly diagnosed breast cancer (<6 months) as compared to those in whom the time elapsed since the diagnosis was more than 6 months. Similarly thrombin generation in the subgroup of newly diagnosed patients (<6 months) on chemotherapy was significantly higher as compared to those on active chemotherapy in whom the time elapsed since the diagnosis of breast cancer was more than 6 months (Table 3, Figure 3).

**Table 3:** **Thrombogram parameters and Pd-MP levels in all patients and in patients on chemotherapy according to the time since the diagnosis (less than 6 months or more)**

	All patients (n = 62)		Patients on chemotherapy (n = 41)	
	0-6 months (n = 27)	>6 months (n = 35)	0-6 months (n = 20)	>6 months (n = 21)
**MRI** ***(nM/min)***	160 ± 60*	153 ± 48	161 ± 55*	148 ± 52
**Peak** ***(nM)***	353 ± 92*	336 ± 70*	362 ± 73*	324 ± 74
***ETP (nM.min)***	1593 ± 340*	1483 ± 332	1627 ± 335*	1402 ± 327
***Pd-MP*** **(/μL)**	10098 ± 7057	9946 ± 9175	10054 ± 7769	9026 ± 7469
***Pd-MP/PS*** ^***+***^ **(/μL)**	9731 ± 6821	9671 ± 8841	9695 ± 7488	8793 ± 7265
***PPL (sec)***	43,9 ± 10,8	43,3 ± 10,1	43,3 ± 12,3	44,7 ± 10,8

Values are mean ± sd. *p < 0,05 versus >6 months.

No significant differences of thrombogram parameters were observed between subgroups of patients with local or regional stage of the disease. The subgroup of patients with metastatic stage was not analysed because it included only 4 newly diagnosed patients (less than 6 months) and 10 patients on active chemotherapy. Thus the impact of the metastatic stage on thrombin generation was confounded.

### Procoagulant platelet-derived microparticles in breast cancer patients

In the control group the concentration of Pd-MP and Pd-MP/PS^+^ was 756 ± 429/μl and 695 ± 361/μL respectively. In the breast cancer group Pd-MP and Pd-MP/PS^+^ were significantly increased (p < 0.001) compared to the control group (Table 2). The concentration of Pd-MP and Pd-MP/PS^+^ was higher than the UNL in 97% and 93% of patients respectively. Accordingly, the PPL clotting time was significantly shorter in patients as compared to the control group (43.5 ± 10 sec versus 72.8 ± 9.9; p = 0.03). The PPL clotting time was significantly correlated with both Pd-MP and Pd-MP/PS^+^ (r^2^ = 0.7; p < 0.0001). In 51 patients (82%) the PPL clotting time was shorter than the LNL of the assay.

### Influence of stage, time and chemotherapy on platelet-derived microparticles

There was no significant difference in Pd-MP or Pd-MP/PS^+^ between the subgroups of patients with local or regional stage of cancer. Patients with metastatic disease had significantly higher levels of Pd-MP and Pd-MP/PS^+^ compared to those with regional stage (Table 2).

The concentration of Pd-MP and Pd-MP/PS^+^ was not influenced by the time since the diagnosis of the breast cancer (Table 3). The stratification of each subgroup according to the administration of chemotherapy did not show any significant difference between the subgroups (Table 3). The PPL clotting time, similarly to Pd-MP, was not influenced by chemotherapy and time since diagnosis but it was significantly shorter in patients with metastatic disease as compared to those with local stage (Table 2).

### D-Dimer levels in breast cancer patients

**Figure 4:**
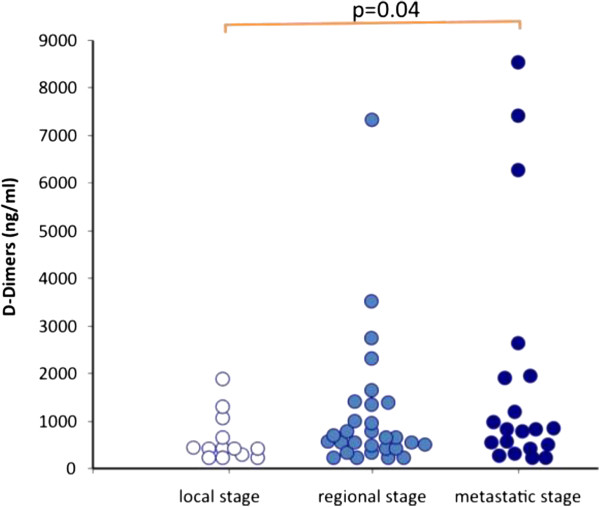
**Impact of the stage of breast cancer on the conentration of D-Dimers.**

The concentration of D-Dimers was significantly increased in cancer patients (1250 ± 1773 ng/ml) compared to the control group (230 ± 50 ng/ml; p < 0.05). The concentration of D-Dimers tended to increase in advanced stages of the disease (Table 2). However no significant difference was observed between the subgroups of patients with local and regional stage (605 ± 499 ng/ml versus 1123 ± 1429 ng/ml; p > 0.05). The concentration of D-Dimers in patients with metastatic stage (1853 ± 2497 ng/ml) was significantly higher as compared to that in patients with local stage (p = 0.049). The concentration of D-Dimers in patients with regional stage was not significantly different as compared to patients with metastatic stage (Figure 4). The analysis of the data from the subgroup of the patients on chemotherapy showed a similar trend of elevation of D-Dimers in parallel with the stage of the cancer.

In patients with localized disease receiving chemotherapy, the concentration of D-Dimers was significantly lower (410 ng/mL, range 220–1230 ng/mL) compared to patients on chemotherapy for metastatic disease (1920 ng/mL, range, 242–6547 ng/mL, p = 0.033). The time since diagnosis of cancer did not show any significant influence on D-Dimer levels in the subgroup of patients having chemotherapy.

In 32 patients (52%) the concentration of D-Dimers in plasma was higher than the age adapted upper normal cut-off level. In 29 patients (46%) the concentration of D-Dimers and at least one parameter of thrombogram were higher than the UNL of the corresponding test.

### Cardiovascular risk factors and markers of cellular and plasma hypercoagulability in breast cancer patients

Thrombin generation, PPL clotting time and the concentration of Pd-MP, PdMP/PS^+^ were not significantly different between the subgroup of patients with at least one risk factor of cardiovascular disease compared to those who did not have any cardiovascular risk factor. In contrast, the concentration of D-Dimers was significantly higher in patients with breast cancer who had at least one cardiovascular risk factor as compared to those who did not have any cardiovascular risk factor (Table 4).

**Table 4:** **Analysis of the impact of cardiovascular risk factors on the biomarkers of cellular and plasma hypercoagulability in patients with breast cancer**

	Total population of patients (n = 62)	
	CV RF (n = 21)	No CV RF (n = 41)
**MRI (mM/min)**	134 ± 51	167 ± 51
**Peak (nM)**	317 ± 89	357 ± 72
**ETP (mM.min)**	1490 ± 346	1552 ± 335
**Pd-MP (/μL)**	8133 ± 6480	10955 ± 8893
**Pd-MP/PS** ^**+**^ **(/μL)**	7896 ± 6328	10599 ± 8552
**PPL (sec)**	42 ± 10	44 ± 11
**D-dimers (ng/ml)**	1190 ± [440–1750]	540 ± [280–830]

CV RF: Cardiovascular risk factors.

Pd-MP: Platelet derived microparticles.

Pd-MP/PS^+^: Platelet derived microparticles expressing phosphatidylserin.

PPL: Procoagulant phospholipid dépendent clotting time.

MRI: Mean rate index of the propagation phase.

ETP: Endogenous thrombin generation.

*p = 0,05 versus No CV RF.

### Correlation of cellular and plasma markers of hypercoagulability with routine hematological and biochemical parameters

Age and BMI of patients did not correlate with any of the studied biomarkers of hypercoagulability. Among thrombogram parameters the Peak and the ETP were significantly correlated with the CRP (r = 0.3; p = 0.028 and 0.019 respectively). The peak was also correlated with the ASAT levels (r = 0.3; 0 = 0.03).

The concentration of D-Dimers was inversely correlated with Hb (r = 0.52; p < 0.0005) and positively correlated with the concentration of transaminases. In addition, alkaline phosphatase was correlated with the concentration of D-Dimers (r = 0.38; p < 0.005). The levels of D-Dimers did not correlate with creatinine, urea and CRP.

The concentration of Pd-MP and Pd-MP/PS^+^ was inversely correlated with Hb (r = −0.3; p = 0.01) and positively correlated with the platelet count (r = 0.3; p = 0.02).

All the other hematological and biochemical parameters did not correlate with thrombin generation parameters and Pd-MP or PPL clotting time. None of thrombin generation parameters was correlated with the concentration of D-Dimers or Pd-MP or with aPTT or PT.

## Discussion

The present study demonstrates that blood hypercoagulability in breast cancer patients is consisted of cellular and plasma components and is characterized by marked increase of procoagulant Pd-MP, enhanced thrombin generation and increased degradation of fibrin. The stage of the disease, the administration of chemotherapy and the time elapsed since the diagnosis, have a significant but variable impact on the cellular and plasma components of hypercoagulability.

Almost all breast cancer patients showed high levels of procoagulant Pd-MP and short PPL clotting time in plasma. Thus, in patients with breast cancer, platelet activation leading to the release of microparticles expressing phosphatidylserine is a principal characteristic of blood borne hypercoagulability. This finding is in accordance with previous studies which showed that breast cancer patients treated with chemotherapy or receiving adjuvant endocrine therapy have increased numbers of Pd-MP and a high microparticle-dependent thrombin generation [28]. Our study shows that the increase of Pd-MP is related to the underlying cancer rather than to the anticancer treatment. Indeed, the stage of the disease has a significant influence on the concentration of the procoagulant Pd-MP and the PPL clotting time. Patients with metastatic disease had significantly higher concentrations of Pd-MP and shorter PPL clotting time compared to those with localized stage. Interestingly, chemotherapy did not induce any significant change on the concentration of Pd-MP or the PPL clotting time. These findings are in accordance with previous studies [29–32] and support the hypothesis that Pd-MP concentration and the PPL clotting time are biomarkers that reflect the close association between the burden of cancer cells and platelets. Whether the release of procoagulant microparticles by platelets stems from the direct interaction of platelets with breast cancer cells or is the consequence of an inflammatory reaction triggered by cancer merits further investigation. In favor of the former hypothesis is that most of the patients in our study showed CRP levels within the normal range. In addition, no correlation was found between Pd-MP or PPL-clotting time and CRP. The concept that platelet activation is a dominant phenomenon in cancer is supported by several recent studies conducted in patients with other types of cancer and may have therapeutic implications in the management of cellular derived hypercoagulability and cancer [33–36].

Platelet-derived microparticles manifested significant procoagulant activity as documented by the almost linear, inverse correlation between the concentration of both Pd-MP and Pd-MP/PS^+^ with the PPL clotting time. However, neither Pd-MP nor PPL-clotting time was correlated with thrombin generation. In our study, thrombogram-thrombinoscope assay was performed in platelet poor plasma using 5 pm of TF and a saturating concentration of procoagulant phospholipids (4 μM). Preliminary experiments from our group showed that in these experimental conditions, the thrombogram assay is not sensitive to the procoagulant activity of microparticles present in the plasma samples (data not shown). Consequently, the two settings of tests describe different components of hypercoagulability; the cellular and the plasma one.

Thrombin generation was significantly increased in patients with breast cancer as compared to the control group. About 76% of patients had the mean rate index (MRI) of the propagation phase of thrombin generation higher than the upper normal limit showing that the increase of thrombin generation is also a major element of the hypercoagulability in breast cancer. The present study documents that a significant cellular and plasma hypercoagulability occurs within the first six months from the diagnosis of breast cancer. Indeed, the increase of thrombin generation was marked in patients diagnosed with cancer within less than 6 months from the inclusion as compared to those to whom the time elapsed since the diagnosis of cancer was longer than 6 months. In addition, thrombin generation was significantly increased in patients with recently diagnosed cancer who were on active chemotherapy as compared to those who were on chemotherapy while the cancer was diagnosed in an interval longer than 6 months from the inclusion. These data lead to the conclusion that during the six months after the diagnosis, the breast cancer cells and chemotherapy are combined stimuli of cellular and plasma hypercoagulability. The Vienna Cancer and Thrombosis Study (CATS), which prospectively evaluated the capacity of biomarkers of hypercoagulability to detect the risk of VTE in cancer patients, showed that high thrombin generation is an independent risk factor for VTE [37]. The data presented herein underline the presence of significant increase of thrombin generation and enhanced platelet activation during the first six months after the diagnosis of breast cancer. This period is characterized in breast cancer patients by a substantially increased risk of VTE [12, 38–43]. The subgroup analysis failed to demonstrate an increase of thrombin generation in patients with metastatic disease versus to those without metastasis; although this was the case for the Pd-MP. This is probably due to the heterogeneity of the subgroup of patients with metastasis. This subgroup was composed by a small number of patients recently diagnosed with cancer and also by a small number of patients on active chemotherapy. As mentioned above, the time since the diagnosis inferior than six months and the administration of chemotherapy are major determinants for the increase of thrombin generation. Consequently the composition of the subgroup of patients with metastatic disease was a confounder for the evaluation of the impact of the stage on thrombin generation. The presence of cardiovascular risk factors was not associated with any significant impact on either thrombin generation or the concentration of Pd-MP and the PPL clotting time. This finding further supports the concept that the increase of Pd-MP concentration and the enhancement of thrombin generation are related to the characteristics of the cancer (i.e. time since diagnosis, stage and active chemotherapy). The poor correlation between Pd-MP and the studied biochemical parameters of inflammation, renal and liver function further enhances the assumption that platelet related hypercoagulability is closely associate with cancer. Thrombin generation showed also a weak correlation with CRP and liver function. In the majority of the patients these markers were within the normal range. Thus plasma hypercoagulability in the studied cohort of breast cancer patients also stems from the interactions of cancer cells with plasma and the impact of chemotherapy rather thanfrom an inflammatory state.

Breast cancer patients also showed enhanced fibrin degradation documented by the significant increase of D-Dimers concentration in patients’ plasma as compared to healthy age-matched individuals. In 59% of patients D-Dimers concentration was higher than the upper normal limit. The D-Dimers concentration in patients with metastatic disease was higher as compared to that in patients with localized cancer. The concentration of D-Dimers did not correlate with the Pd-MP concentration, the PPL clotting time or the enhancement of thrombin generation. In 46% of patients a combined increase of D-Dimers and thrombin generation was observed documenting a dissociation between these biomarkers which apparently measure different aspects of the interactions between breast cancer cells, blood coagulation, platelets and fibrinolysis. The elevation of D-Dimers, particularly in the advanced stage of the disease, is in accordance with previous studies which demonstrated that the augmentation of D-Dimers might be in part a reflection of ongoing fibrinogen metabolism within the actively remodeled tumor stroma [44]. The increase of D-Dimers is related to the clinically measured growth rate of breast cancer, the tumor volume, the progression rate and the survival in patients with metastatic breast cancer [44]. Tumor cells possess strong procoagulant activities that induce local activation of the coagulation system and deposition of fibrin, which has an important role in the formation of tumor stroma and hematogenous spread of tumor cells [45]. The Vienna Cancer and Thrombosis Study (CATS) showed that enhanced activation of coagulation and fibrinolysis, as reflected by high levels of D-dimers, is independently associated with an unfavorable prognosis in patients with solid cancers and is not necessarily mediated by the increased risk of VTE [22]. Our data, being in accordance with these evidence allow to conclude that in breast cancer patients the D-Dimers is a biomarker which is related with a global biological activity of cancer cells which is not restricted to plasma hypercoagulability. Noteworthy in our study, the presence of cardiovascular risk factors was linked with a significant increase of the concentration of D-Dimers revealing that the D-Dimers is a less specific biomarker for cancer induced hypercoagulability.

The identification of breast cancer patients at risk of VTE and the optimization of thromboprophylaxis is a puzzling exercise because VTE risk varies according to the type of cancer and is potentially influenced by its evolution, the histology and the localization of the cancer, the duration and the intensity of chemotherapy or other adjuvant treatments. The development of Risk Assessment Models (RAM) for VTE risk stratification adapted for cancer patients and their prospective clinical validation is required. Such a RAM - focused on cancer patients receiving chemotherapy - has been proposed and prospectively validated by Khorana [16]. This model includes some clinical risk factors such as the site of cancer, the body mass index, the increased pre-chemotherapy platelet and leukocyte counts. However, other variables related to the malignant disease which contribute to the VTE risk (i.e. the stage, the type of anticancer therapy etc.) are lacking. In addition, the Khorana RAM does not include breast cancer. The data from our study show that patients with breast cancer show cellular and plasma blood borne hypercoagulability which is influenced by the stage of the disease, the time elapsed from the diagnosis and the administration of chemotherapy. The weighted incorporation of the studied biomarkers of cellular and plasma hypercoagulability in association with the clinical characteristics of breast cancer and the other intrinsic risk factors for VTE present in patients could formulate a new specific RAM for breast cancer patients. To this aim a prospective studied is needed.

## Conclusions

The present study identified the most appropriate biomarkers for the diagnosis of blood borne hypercoagulability related to breast cancer. The concentration and the procoagulant activity of Pd-MP are interconnected with the biological activity and the overall burden of cancer cells. Assessment of thrombin generation is related with both the procoagulant characteristics of breast cancer andthe procoagulant effect of anticancer treatment administration within 6 months after the diagnosis of the disease. Thus the weighted incorporation of the biomarkers of cellular and plasma hypercoagulabilty in risk assessment models for VTE might lead to the elaboration of a breast cancer specific RAM with improved predictive value.

## Abbreviations

aPTT: Activated partial thromboplastin time
ETP: Endogenous thrombin potential
LNL: Lower normal limit
MRI: Mean rate index
Pd-MP: Platelet derived microparticles
Pd-MP/PS^+^: Platelet derived microparticles expressing phosphatidylserin
PPL: Procoagulant phospholid dependent clotting time
PPP: Platelet poor plasma
PT: Prothrombin time
RAM: Risk Assessment Model
TF: Tissue factor
TNM: Tumor, node, metastases
UNL: Upper normal limit
VTE: Venous thromboembolism.
